# Considering additive effects of polypharmacy: Analysis of adverse events in geriatric patients in long-term care facilities

**DOI:** 10.1007/s00508-020-01750-6

**Published:** 2020-10-22

**Authors:** Monika Lexow, Kathrin Wernecke, Gordian L. Schmid, Ralf Sultzer, Thilo Bertsche, Susanne Schiek

**Affiliations:** 1 Drug Safety Center, University Hospital of Leipzig, Leipzig University, Brüderstr. 32, 04103 Leipzig, Germany; 2 Dept. of Clinical Pharmacy, Leipzig University, Brüderstr. 32, 04103 Leipzig, Germany; 3 Dept. of General Practice, Medical Faculty, University of Leipzig, Philipp-Rosenthal-Str. 55, 04103 Leipzig, Germany; 4 Sana Geriatric Hospital Zwenkau, Pestalozzistr. 9, 04442 Zwenkau, Germany

**Keywords:** Nursing homes, Side effects, Aged, Adverse drug reactions, Naranjo algorithm

## Abstract

**Background:**

Potential additive effects of polypharmacy are rarely considered in adverse events of geriatric patients living in long-term care facilities. Our aim, therefore, was to identify adverse events in this setting and to assess plausible concomitant drug causes.

**Methods:**

A cross-sectional observational study was performed in three facilities as follows: (i) adverse event identification: we structurally identified adverse events using nurses’ interviews and chart review. (ii) Analysis of the concomitantly administered drugs per patient was performed in two ways: (ii.a) a review of summary of product characteristics for listed adverse drug reactions to identify possible causing drugs and (ii.b) a causality assessment according to Naranjo algorithm.

**Results:**

(i) We found 424 adverse events with a median of 4 per patient (range 1–14) in 103 of the 104 enrolled patients (99%). (ii.a) We identified a median of 3 drugs (range 0–11) with actually occurring adverse events listed as an adverse drug reaction in the summary of product characteristics. (ii.b) Causality was classified in 198 (46.9%) of adverse events as “doubtful,” in 218 (51.2%) as “possible,” in 7 (1.7%) as “probable,” and in 1 (0.2%) adverse event as a “definitive” cause of the administered drugs. In 340 (80.2%) of all identified adverse events several drugs simultaneously reached the highest respective Naranjo score.

**Conclusion:**

Patients in long-term facilities frequently suffer from many adverse events. Concomitantly administered drugs have to be frequently considered as plausible causes for adverse events. These additive effects of drugs should be more focused in patient care and research.

## Introduction

Geriatric patients in long-term care (LTC) facilities are multimorbid and, therefore, suffer from many (non)specific symptoms and geriatric syndromes [1]. Disease-related symptoms should be distinguished from adverse drug reactions (ADR) that result from drug therapy [2]. The latter can lead to hospital admissions and have a considerable impact on morbidity and mortality with high costs in the health care system [3–5]. Polypharmacy makes a significant contribution to the clinical consequences deriving from ADRs in geriatric patients [6]. For this reason, a structured analysis of adverse events (AE) and drug-related causes in these patients is of high interest for routine care.

Distinguishing whether an observed AE was caused by a disease (i.e. symptom) or by a drug (ADR) poses a challenge for healthcare professionals [7, 8]. The correct attribution is required for appropriate treatment strategies but can result only from structured detection, analysis and classification. Geriatric patients are frequently cognitively impaired or suffer from speech or hearing disorders. Hence, information provided by the patients is often insufficient. In LTC facilities, therefore, chart documentation and nurses’ interviews are the most valuable sources for AE detection [9, 10]. So far, no specific method exists to analyze and classify AE in LTC facility residents with polypharmacy. The Naranjo algorithm has previously been used for causality assessment in this collective [11, 12]. It allows a detailed assessment of every detected AE and every single administered drug. This algorithm provides further information on drug-related causes when combined with established methods for patient safety, such as drug-drug interactions and potentially inappropriate medications [13].

Causality scores like the Naranjo score, however, do not consider simultaneously contributing drugs. For some ADRs, it has been shown that the number of specific drugs causes their clinical manifestations. For example, patients are exposed to an increased risk of falling when they take two or more drugs which increase the risk of falling [14]. Concerning anticholinergic ADRs, it is common to calculate an anticholinergic burden to quantify the risk for an adverse outcome [15]. Little is known, however, about additive drug effects in other events. Therefore, data about potential additive effects in this vulnerable patient collective are of great interest for routine care.

The aim of this study was to identify AEs occurring in LTC facility patients and to assess plausible concomitant drug causes.

## Patients, material and methods

### Definitions

We defined an AE as an outcome that occurs while a patient is taking a drug, but is not or not necessarily attributable to it and an ADR as an appreciably harmful or unpleasant reaction, resulting from an intervention related to the use of a medicinal product [16]. We used the term drug not only for the effective substance but for the whole product prescribed in the medication chart of the patient. A drug therefore could contain more than one active substance. We considered all drugs administered to the patient during the acquisition period. Continuous and on-demand medications were assessed separately because the temporal relationship between AE and administration of the drug could be different in that case.

### Participants and setting

We conducted a cross-sectional observation study in three LTC facilities in Germany. After written informed consent of the residents or their legal representative and the responsible general practitioner, residents in the participating LTC facilities were enrolled in the study. We included residents of facilities with different ownerships (welfare, municipal or private associations) to approach a representative sample of 100 residents. Inclusion criteria were: informed consent, age ≥65 years, long-term/chronic medicines ≥3 and multimorbidity with ≥3 comorbidities at the time of recruitment, more than 8 weeks stay in the LTC facility, and a life expectancy of more than 6 months according to nurses’ present information. The study was conducted over a time period of 10 months.

### Study design and data collection

We conducted a structured analysis of AEs.

#### (i) AE identification

We used two complement sources of information for our structured data collection: Firstly, an interview about individual AEs with nurses involved in daily care and, secondly, a review of residents’ records (electronic and chart documentation, laboratory values) for documented events and their temporal occurrence. To ensure standardized identification of AEs, a checklist of events was applied to both methods. The listed events comprised the most relevant AEs or ADRs for geriatric patients and LTC residents based on the literature [17–19]: blackened stool, bleeding/hematoma, confusion/disorientation, constipation, depression/anxiety, diarrhea, dizziness/vertigo, dry mouth, ear disorders, eye disorders, falls, hallucination, hyperglycemia/hypoglycemia, hyperhidrosis, hyperkalemia/hypokalemia, hypernatremia/hyponatremia, nausea, pain, restlessness, skin disorders/pruritus, insomnia, urinary incontinence, vomiting (in alphabetical order). Additional relevant reported or documented events were collected as well. We considered reported and documented symptoms during a time period of the prior 30 days (considered as 1 resident month) for new and continuous symptoms. Data collection was performed at two measurement points per patient at intervals of 6–8 weeks by two clinical pharmacists. All detected AEs and the corresponding system organ class were classified based on the common terminology criteria for adverse events (CTCAE) [20].

#### (iia) Review of summary of product characteristics

We systematically collected data from the medical charts (continuous drugs, on demand drugs and their frequency of use, date of first administration). We checked all summaries of product characteristics (SmPCs) of the actually administered drugs for listed ADRs. A drug would be considered as “potentially causing” if the listed ADR in the SmPC represented a synonym for the detected AE or possibly caused it (e.g. dizziness in cases of falls). For the analysis of additive effects, we counted the number of potentially causing drugs. Prescribed drugs were characterized by their code in the anatomical therapeutic chemical classification system (ATC code).

#### (iib) Causality assessment according to the Naranjo algorithm

We used the Naranjo algorithm for causality assessment. All further relevant information, such as the duration of the AE, underlying diseases, clinical consequences (e.g. from hospital report), laboratory values, and patient-specific conditions were collected and used to determine the Naranjo score. The most likely associated drugs were the ones that reached the highest Naranjo score concerning the single analyzed AE. Naranjo distinguishes between definitive with a total score ≥9, probable with 5 < total score < 8, possible with 1 < total score < 4 and doubtful with a total score ≤ 0 [21, 22].

Inconclusive evaluations in all steps (i, ii.a, and ii.b) were discussed and finalized by mutual agreement in an expert panel. This panel consisted of four experienced clinical pharmacists.

### Statistical analysis

To ensure comparable patient parameters between the three LTC facilities independent of the allocation to a single facility, main patient parameters were statistically analyzed. For this purpose, a Kruskal Wallis test with pairwise comparison was performed. Analyzed parameters were age, gender, number of diagnoses and number of continuous and on demand drugs, as well as the number of AEs in the patients and the maximum Naranjo score per patient. The data analysis was performed using IBM SPSS Statistics Version 25.0 (IBM Corporation, Armonk, NY, USA) and Microsoft Office Excel 2013 (Microsoft Corporation, Redmond, WA, USA). *P*-values ≤ 0.05 were considered as statistically significant.

## Results

### Patient characteristics

In the participating parts of the LTC facilities, 182 patients were potentially available for the study and 154 met the inclusion criteria. From these, 104 patients or their legal guardian gave their informed consent as well as their responsible physician and were enrolled in the study. Patients were mostly female (72.1%) and in median 86 (range: 66–101) years old (Table 1). Patients did not differ between the three LTC facilities according to the following parameters: age (*p* = 0.311), gender (*p* = 0.684), number of diagnoses (*p* = 0.070) and number of continuous (*p* = 0.629) and on demand drugs (*p* = 0.911).

**Table 1:** Characteristics of patients included in the study with frequency of documented diagnoses, main ATC classes and main active substances

Characteristics	Value
Patients, total, *n*	104
Patients in facility of welfare ownership, *n* (%)	34 (32.7%)
Patients in facility of municipal ownership, *n* (%)	30 (28.8%)
Patients in facility of ownership by private association, *n* (%)	40 (38.5%)
Female, *n* (%)	75 (72.1%)
Length of residence (months), median (Q25/Q75; min–max)	31 (12/63; 1–414)
Age (years), median (Q25/Q75; min–max)	86 (78/90; 66–101)
Documented diagnoses, median (Q25/Q75; min–max)	15 (10/21; 3–35)
No. of continuous drugs, median (Q25/Q75; min–max)	8 (6/10; 2–18)
No. of on demand medication, median (Q25/Q75; min–max)	2 (1/3; 1–6)
*Documented diagnosis*^a^	
Hypertension, *n* (%)	82 (78.8%)
Dementia, *n* (%)	69 (66.3%)
Diabetes, *n* (%)	41 (39.4%)
Heart failure, *n* (%)	32 (30.8%)
Atrial fibrillation, *n* (%)	32 (30.8%)
Renal failure, *n* (%)	24 (23.1%)
Osteoporosis, *n* (%)	19 (18.3%)
Stroke, *n* (%)	17 (16.3%)
*Main ATC classes*^b^	
C (cardiovascular system), *n* (%)	236 (28.7%)
*N* (nervous system), *n* (%)	216 (26.3%)
A (alimentary tract and metabolism), *n* (%)	164 (20.0%)
B (blood and blood-forming organs), *n* (%)	68 (8.3%)
H (systemic hormonal preparations, excluding sex hormones and insulins), *n* (%)	27 (3.3%)
*Main active substances*^b^	
Torasemide, *n* (%)	47 (5.7%)
Pantoprazole, *n* (%)	40 (4.9%)
Ramipril, *n* (%)	35 (4.3%)
Acetylsalicylic acid, *n* (%)	33 (4.0%)
Metoprolol, *n* (%)	23 (2.8%)

*ATC* anatomical therapeutic chemical/defined daily dose classification, *Q25/Q75* first and third quartile

^a^Order is based on the most relevant diagnoses found in literature data to geriatric patients

^b^According to the documented continuous drugs

#### (i) AE identification

From a total of 104 patients, at least 1 AE was identified in 103 (99.0%). We identified 424 AEs, with a detected median of 4 (Q25/Q75: 2/5, range 1–14) AEs per patient, which equals 2.05 AEs per resident month. The identified AEs and the number of affected patients are shown in Table 2. The system organ classes renal and urinary disorder (87 patients), gastrointestinal disorder (43 patients), skin and subcutaneous tissue disorders (37 patients) were most common in our patient collective. Altogether, 72 different AE categories were detected, 185 AEs were identified in the patient records and 195 AEs by the nurses’ interviews, with 44 AEs in concordance of both methods. We found a significant difference in the detected number of AEs between the observed LTC facilities (*p* = 0.020). Following the pairwise comparison, we only found differences between the municipal LTC facility with 3 (Q25/Q75: 2/4) AEs and the private LTC facility with a median of 4 (Q25/Q75: 3/6.25) AEs (*p* = 0.022).

**Table 2:** Identified adverse evnts (*n* = 424) according to CTCAE and affected patients (*n* = 104)

System organ class	Number of identified AEs, *n* (%)	Affected patients, *n* (%)	AE with number of affected patients^a^ (*n*)
Renal and urinary disorders	88 (20.8)	87 (83.7)	Urinary incontinence (87), urinary tract pain (1)
Gastrointestinal disorders	55 (13.0)	43 (41.3)	Constipation (22), vomiting (16), diarrhea (10), blackened stools (3), nausea (2), lower gastrointestinal bleeding (1), periodontal disease (1)
Psychiatric disorders	55 (13.0)	35 (33.7)	Confusion (21), restlessness (12), defensive behavior (8), insomnia (5), depression (4), anxiety (2), hallucinations (1), personality change (1), psychiatric disorders—other specify (1)
Skin and subcutaneous tissue disorders	50 (11.8)	37 (35.6)	Intertrigo (9), dry skin (7), hyperhidrosis (7), skin ulceration (6), local redness (5), pruritus (4), purpura (4), skin and subcutaneous tissue disorders—other specify (3), skin induration (1), urticaria (2), alopecia (1), angioedema (1)
Metabolism and nutritional disorders	41 (9.7)	27 (26.0)	Hyperglycemia (26), hypoglycemia (15)
Musculoskeletal and connective tissue disorders	40 (9.4)	33 (31.7)	Arthralgia (14), pain in extremity (12), back pain (6), arthritis (4), musculoskeletal and connective tissue disorders—other specify (3), general muscle weakness (1)
Nervous system disorders	39 (9.2)	31 (29.8)	Dizziness (11), somnolence (10), headache (4), syncope (3), ataxia (2), cognitive disturbance (2), paresthesia (2), depressed level of consciousness (1), lethargy (1), neuralgia (1), seizure (1), spasticity (1)
Injury, poisoning and procedural complications	14 (3.3)	14 (13.5)	Fall (14)
Vascular disorders	11 (2.6)	11 (10.6)	Hematoma (10), flushing (1)
Infections and infestations	8 (1.9)	7 (6.7)	Skin infection (4), vulval infection (2), conjunctivitis infective (1), stoma site infection (1)
General disorders and administration site conditions	7 (1.7)	7 (6.7)	Edema limbs (3), pain (3), fatigue (1)
Respiratory, thoracic and mediastinal disorders	7 (1.7)	6 (5.8)	Dyspnea (4), cough (1), epistaxis (1), respiratory, thoracic and mediastinal disorders—other specify (1)
Ear and labyrinth disorders	3 (0.7)	3 (2.9)	Hearing impaired (2), tinnitus (1)
Cardiac disorders	2 (0.5)	2 (1.9)	Chest pain—cardiac (1), palpitations (1)
Eye disorders	2 (0.5)	1 (1.0)	Blurred vision (1), glaucoma (1)
Investigations	2 (0.5)	2 (1.9)	Weight gain (1), weight loss (1)

*AE(s)* adverse event(s), *CTCAE* common terminology criteria for adverse events

^a^Multiple categories per patient possible

#### (ii.a) Review of summary of product characteristics

**Fig. 1:**
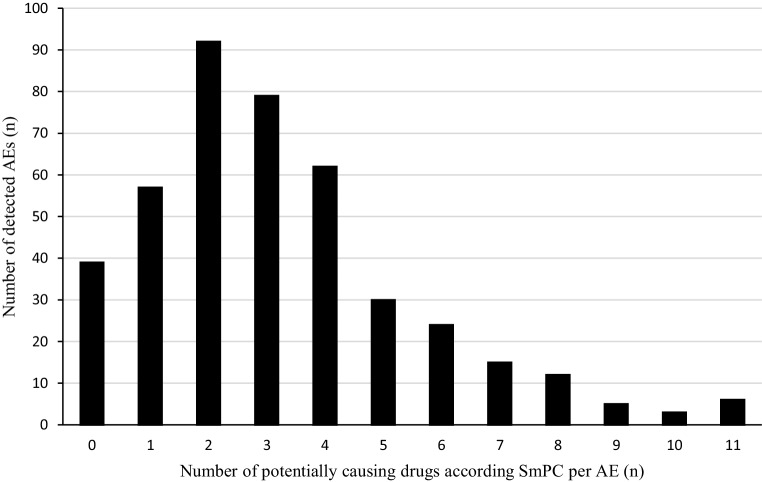
Number of detected adverse events versus number of potentially causing drugs according Summary of Product Charactetistics. *AE(s)* adverse event(s), *SmPC* summary of products characteristics

**Fig. 2:**
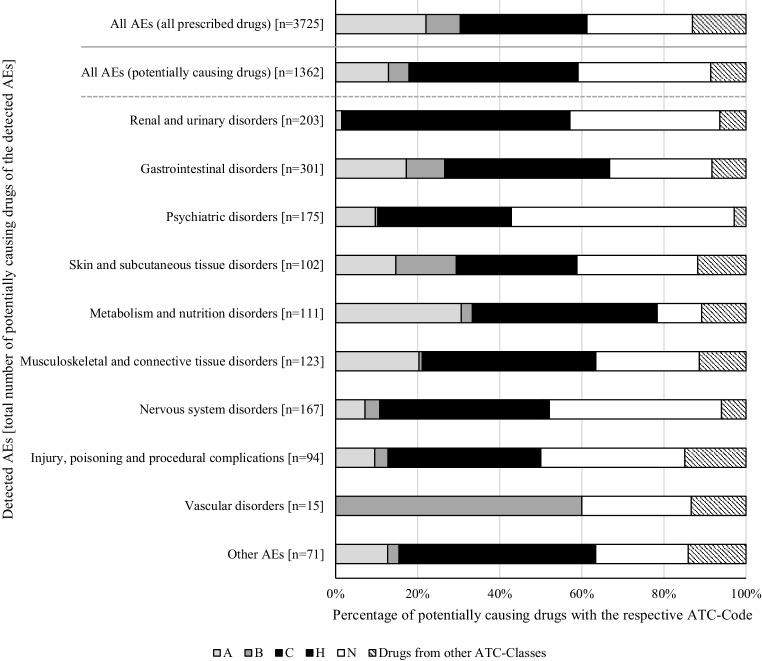
Potentially causing drugs according Summary of Product Characteristics differentiated into the ATC classes for the most frequently detected system organ classes. *AE(s)* adverse event(s), *ATC* anatomical therapeutic chemical/defined daily dose classification, *CTCAE* common criteria for the terminology of adverse events, *SmPC* summary of products characteristics. ATC classes: A: alimentary tract and metabolism, B: blood and blood forming organs, C: cardiovascular system, H: systemic hormonal preparations, excluding sex hormones and insulins, N: nervous system

To analyze the concomitantly administered drugs, we assessed 3725 combinations of AEs and corresponding drugs. For this analysis five drug/AE pairs had to be excluded because no information from the SmPC was available (moisturizing eye drops, medical device). Considering every identified AE, patients had a median of 3 potentially causing drugs according to the SmPC, with a range from 0 to 11 drugs (Q25/Q75: 2/4; details in Fig. 1). The most frequently (*n* ≥ 10) detected AEs and the affected system organ classes are shown in Table 3. The ATC classes prescribed most often (C, N, A, B, H) were frequently among the potentially causing drugs for the most common system organ classes (Fig. 2).

**Table 3:** Median number of potentially causing drugs according Summary of Product Charactersitics and corresponding Naranjo Score per patient (*n* = 104) for the most frequently detected (≥10) adverse events (AEs) and for their corresponding System organ classes (all 424 detected AE included)

System organ class and ... most frequent AE	Number (*n*)	Median number of potentially causing drugs per patient [range]	Median Naranjo score [range]
Renal and urinary disorders	88	2 [0–5]	0 [−1–2]
... Urinary incontinence	87	2 [0–5]	0 [−1–2]
Gastrointestinal disorders	55	5 [0–11]	2 [0–4]
... Constipation	22	5 [0–10]	0 [0–3]
... Vomiting	16	7.5 [2–10]	2 [0–4]
... Diarrhea	10	4.5 [2–11]	3 [0–3]
Psychiatric disorders	55	3 [0–7]	0 [−1–9]
... Confusion	21	3 [1–7]	1 [0–8]
... Restlessness	12	3 [0–4]	0 [−1–3]
Metabolism and nutrition disorders	41	3 [0–5]	1 [0–5]
... Hyperglycemia	26	3 [0–4]	1 [0–1]
... Hypoglycemia	15	3 [1–5]	4 [2–5]
Musculoskeletal and connective tissue disorders	40	3 [0–8]	2 [−1–3]
... Arthralgia	14	3 [0–6]	1 [−1–3]
... Pain in extremity	12	2 [1–8]	2 [0–3]
Nervous system disorders	39	4 [0–10]	1 [−1–7]
... Dizziness	11	5 [1–10]	2 [0–7]
... Somnolence	10	4 [3–7]	3 [0–5]
Injury, poisoning and procedural complications	14	6 [3–11]	2 [0–3]
... Fall	14	6 [3–11]	2 [0–3]
Vascular disorders	11	1 [0–3]	1 [0–3]
... Hematoma	10	1 [0–3]	0.5 [0–2]

*AE(s)* Adverse Event(s), *SmPC* Summary of product characteristics

#### (ii.b) Causality assessment according to the Naranjo algorithm

All 3730 drug/AE pairs were included in the causality assessment. From the 424 identified AEs, 198 (46.9%) were classified as ADR with “doubtful”, 218 (51.2%) “possible”, 7 (1.7%) “probable”, and 1 (0.2%) “definitive” cause (Table 4). We found no significant differences in the maximum Naranjo scores per patient between the three LTC facilities (*p* = 0.964). On the basis of 424 detected AEs, only 1 drug in 84 AEs (19.8%) and several drugs in 340 AEs (80.2%) reached the highest score (Table 4). According to Naranjo these need to be considered as the most likely causing drug(s).

**Table 4:** Results of the adverse event drug causality assessment according to the Naranjo algorithm

Naranjo Score per AE	Identified AE [*n*]	Number of affected patients^a^, [*n*]	Classification	Identified AE per class, *n* (%)	Number of AE with one/several highest Naranjo drug(s) [*n*]
−1	22	17	Doubtful	199 (46.9)	38/161
0	177	92			
1	68	48	Possible	217 (51.2)	38/179
2	90	51			
3	41	30			
4	18	18			
5	3	3	Probable	7 (1.7)	7/0
6	2	2			
7	1	1			
8	1	1			
9	1	1	Definitive	1 (0.2)	1/0

*AE(s)* adverse event(s)

^a^Several AEs per patient possible

The probable and definitive ADRs were as follows: angioedema (severity grade according to CTCAE 4) induced by enalapril, urticaria (grade 2) induced by amoxicillin and clavulanic acid, hypoglycemia (grade 1) induced by insulin glargine, paresthesia (grade 2) induced by tapentadol and a complex case of occurring hallucinations (grade 3) in combination with confusion (grade 3), dizziness (grade 3) and somnolence (grade 3, in total 4 detected AEs) which were attributable to digitoxin (highest Naranjo score). In this case, the patient also received high doses of oxycodone and duloxetine. It can be seen as a mixed intoxication based on the hospital report. In the algorithm, digitoxin reached a one-point higher score than oxycodone/duloxetine because measurement of the increased blood level was available only for digitoxin.

All of the detected AEs and ADRs were managed adequately by the nurses, for example, by informing a physician or arranging a hospital admission for the affected patient. Thus, no further action was required due to this study.

## Discussion

In our study we addressed AEs in geriatric patients living in LTC facilities. We assessed which type of AEs occurred and also investigated potential additive effects of polypharmacy. With nearly all (99%) patients affected by AEs, we demonstrated the relevance of this topic. We found the identified AEs potentially caused by up to 11 different administered drugs. The Naranjo algorithm showed at least possible drug causes in half of these AEs. Thereby, multiple drugs were equally likely involved 80% of the time. Our results point out that AEs should be systematically recorded in routine practice in LTC facilities. In order to prevent ADRs, additive effects need to be considered in any strategies developed.

### Prevalence of AE and ADR in LTC residents

More than half of our identified AEs could be associated with drug use. Our rate of probable and definitive ADRs was similar to other studies in the LTC setting (0.04 vs. up to 0.10 ADRs per observed resident month), although studies should be compared with caution [9, 23]. Nevertheless, the causality assessment leaves us with a high number of possible ADRs. Especially for AEs which were ongoing for a longer period, causality assessment was challenging in the routine setting. Information to evaluate the exact temporal connection between AE and drug use was frequently missing and therefore could have led to lower Naranjo scores. To resolve this problem, a regular and structured routine assessment of AEs and potentially causing drugs might increase the chance to identify ADRs and protect patients from the consequences.

Our overall rate of identified AEs was higher than results seen in other studies (2.05 AEs vs 0.03–0.12 per observed resident month) [9, 23]. This indicates that we identified a noticeable amount of the general symptom burden of LTC residents that results from underlying diseases or age-related changes. This is consistent with the fact that incontinence, pain, sleep disorders and psychopathological symptoms are widely found in LTC residents [24]. Therefore, a regular routine AE assessment can support ADR detection as well as structured symptom evaluation.

### Additive effects of polypharmacy

The suspected AE was listed as an ADR in the respective SmPC in a median of 3 and up to 11 administered drugs per patient. In 80% of all identified AEs, various drugs reached the highest Naranjo score simultaneously. This means that they were equally likely to cause the AE. This coincidence can increase the chance of AE occurrence independently from single causality scores. This result also raises the question whether ADRs resulting from additive effects have been underestimated. In cases with “probable” or “definitive” ADRs (Naranjo ≥5), we found results from only one drug with the highest Naranjo score; however, in four of these AEs, the drug with the highest Naranjo (digitoxin) was only part of a mixed intoxication with duloxetine and oxycodone based on the hospital report for the affected patient. In this case, the sole consideration of the causality assessment could mask an additive effect of at least 3 concomitantly given drugs. This shows that additive effects need to be considered in every detected AE independently from the single causality. Besides ADRs from well-known drug classes (e.g. vascular ADRs from drugs affecting blood and blood-forming organs), in a substantial amount of AEs, we found involvement of varying ATC-classes that are less familiar (e.g. nervous system ADRs in drugs affecting the cardiovascular system). This underlines the complexity of geriatric patient treatment and the need for interdisciplinary medication reviews that include an assessment of drug-related problems, such as drug-drug interactions, potentially inappropriate medication, as well as ADRs [25, 26]. In routine care, however, potential additive effects are often not taken into account. In particular, new and unclear symptoms could be misinterpreted as new diseases and sometimes even lead to prescribing cascades [27, 28].

### Implications for practice

Firstly, our study shows the need for a good data base and a regular routine assessment of AEs that occur in LTC facilities. We found a very low concordance rate of only 10% between AEs detected in nurses’ interviews and those mentioned in the patient record analysis. This demonstrates the potential of information loss in LTC facilities due to heterogeneous and incomplete AE documentation [29]. It also indicates the potential of recall bias in the nurses. The identification of every occurring AE allows a better assessment of simultaneously occurring events. We found in our study, for example, a combination of vomiting and diarrhea that indicated an infection rather than an ADR. Furthermore, the information on patients’ current symptoms contributes to appropriate proposals for medication changes in cases of identified ADRs.

Secondly, our results support the development of strategies with improved consideration of the additive effects of polypharmacy. Combining an AE assessment with structured medication reviews improves the drug cause analysis of AEs as well as the detection and interpretation of drug-related problems. Ongoing prospective evaluation of AEs and potential drug-related causes contributes to prevent patients from experiencing negative events. This process could be further accelerated by electronic assistance. Electronic documentation of AEs and computer-assisted signal detection of ADRs can support problem solving in a narrow timeframe since physicians and pharmacists are usually not permanently present in the LTC facilities [11, 30]. Database-supported comparison of the events with patients’ medication can assist pharmacists in a comprehensive medication review. Currently, such electronic solutions are rarely used in the LTC setting in Germany. They could also support future research by providing information on the additive effects of various combined drugs and underlying diseases.

Thirdly, our data suggest the need for improvement in interdisciplinary communication in LTC facilities. In interprofessional teams with nurses, pharmacists and physicians, systematic information about AEs, medication reviews and actual health conditions could be transmitted more effectively in patient-orientated practice.

## Conclusion

Nearly every long-term care resident suffered from adverse events (AEs), with half of them at least possibly caused by drugs. In four fifths of these AEs, several concomitantly given drugs were equally associated causes. Therefore, potential additive effects need to be considered independently from single causality and should be more focused in further research. A routinely implemented structured search for AEs and additive effects of polypharmacy contributes to medication reviews and interdisciplinary collaboration and will help to meet the needs of this complex patient collective and to protect them from negative consequences.
